# Glucocorticoid receptor beta increases migration of human bladder cancer cells

**DOI:** 10.18632/oncotarget.8430

**Published:** 2016-03-27

**Authors:** Lucien McBeth, Assumpta C. Nwaneri, Maria Grabnar, Jonathan Demeter, Andrea Nestor-Kalinoski, Terry D. Hinds

**Affiliations:** ^1^ Center for Hypertension and Personalized Medicine, Department of Physiology and Pharmacology, University of Toledo College of Medicine, Toledo, OH 43614, USA; ^2^ Advanced Microscopy and Imaging Center, Department of Surgery, University of Toledo College of Medicine, Toledo, OH 43614, USA

**Keywords:** glucocorticoid receptor, GR, GR alpha, GR beta, glucocorticoids

## Abstract

Bladder cancer is observed worldwide having been associated with a host of environmental and lifestyle risk factors. Recent investigations on anti-inflammatory glucocorticoid signaling point to a pathway that may impact bladder cancer. Here we show an inverse effect on the glucocorticoid receptor (GR) isoform signaling that may lead to bladder cancer. We found similar GRα expression levels in the transitional uroepithelial cancer cell lines T24 and UMUC-3. However, the T24 cells showed a significant (*p* < 0.05) increased expression of GRβ compared to UMUC-3, which also correlated with higher migration rates. Knockdown of GRβ in the T24 cells resulted in a decreased migration rate. Mutational analysis of the 3′ untranslated region (UTR) of human GRβ revealed that miR144 might positively regulate expression. Indeed, overexpression of miR144 increased GRβ by 3.8 fold. In addition, miR144 and GRβ were upregulated during migration. We used a peptide nucleic acid conjugated to a cell penetrating-peptide (Sweet-P) to block the binding site for miR144 in the 3′UTR of GRβ. Sweet-P effectively prevented miR144 actions and decreased GRβ expression, as well as the migration of the T24 human bladder cancer cells. Therefore, GRβ may have a significant role in bladder cancer, and possibly serve as a therapeutic target for the disease.

## INTRODUCTION

Bladder cancer was the fourth most prevalent cancer in men, and fifth overall in 2015 [1]. Recently, glucocorticoids (GCs) have been used in bladder cancer for their protective properties against the toxic effects of chemotherapy [2]. GCs may cause resistance to cisplatin, which is a treatment commonly used for bladder cancer [3, 4]. The GC receptor (GR) is expressed as different isoforms, GRα and GRβ, which are a result of alternative splicing of a single gene [5–9]. GCs bind and activate the ligand-binding GR isoform, GRα, which is a transcription factor that increases genes involved in cell cycle arrest and apoptosis [10–14]. GRβ lacks the ligand-binding domain for GCs [7, 9], and has been shown to be inhibitory to GRα [7, 8, 15–17]. A higher total GR expression has been correlated with a better prognosis in bladder cancer [3, 18]. However, the specific roles of GRα or GRβ in bladder cancer are unknown.

Recent work has shown a conundrum, in that GCs can suppress bladder cancer invasion, but also induce proliferation [18, 19]. GCs are commonly used to inhibit growth in hematological cancers [20] and solid tumors [21]. Long-term GC treatment can increase the risk of bladder cancer, possibly through immunosuppression [2, 4], or by causing GC resistance through elevated GRβ [4, 9]. The later remains to be elucidated. We have recently shown that GRβ can suppress the phosphatase and tensin homolog deleted on chromosome 10 (PTEN) expression and increase Akt1 guided proliferation [8]. Furthermore, GRβ has been shown to be involved in the migratory process of astrocytes and the development of glioblastoma [22]. Longui *et al.* showed that the effectiveness of GCs in patients was reduced with a lower GRα/GRβ ratio [20]. Factors that regulate the expression of GRα or GRβ may influence the response to GCs, and possibly mediate growth. GC resistance in sepsis has been shown to be affected by microRNA 124 (miR124), which down-regulated GRα, causing increased immune cell growth [23].

It has been previously demonstrated that a naturally occurring mutation in the AUUA motif of the 3′ untranslated region (UTR) of GRα and GRβ results in increased mRNA stability and protein expression [24]. Targeting of the 3′ UTR of genes by miRNAs may alter mRNA stability, which has been recently recognized to be involved in processes that regulate cancer development or progression [25–27]. Some miRNAs have been proposed as biomarkers to detect and predict the severity of bladder cancer [28–31]. The miRNAs that may regulate bladder cancer proliferation may be of importance, which was shown by miR125b targeting of the E2F3 transcription factor [32], a tumor suppressor that regulates the cell cycle. Furthermore, miR145 and miR133a decreased bladder cancer aggressiveness by targeting fascin actin-bundling protein 1 (FSCN1) [33], which binds β-catenin to increase motility and invasion. Higher-grade bladder tumors have been shown to express elevated miR144 [34], which has also been shown to promote cell proliferation in nasopharyngeal carcinoma [35]. However, the involvement of miRNAs and their regulation of GRα or GRβ in bladder cancer development or progression are unknown.

In this investigation, we show that GRβ enhanced migration of human bladder cancer cells. We found three potential miRNA target sites in the 3′ UTR of human GRβ and show that miR144 positively affected human GRβ expression. Additionally, we show that blocking the binding site of miR144 in the 3′ UTR of human GRβ inhibited expression, and, as a result, decreased migration of bladder cancer cells.

## RESULTS

### GRβ & GRα in human bladder cancer cells

**Figure 1:**
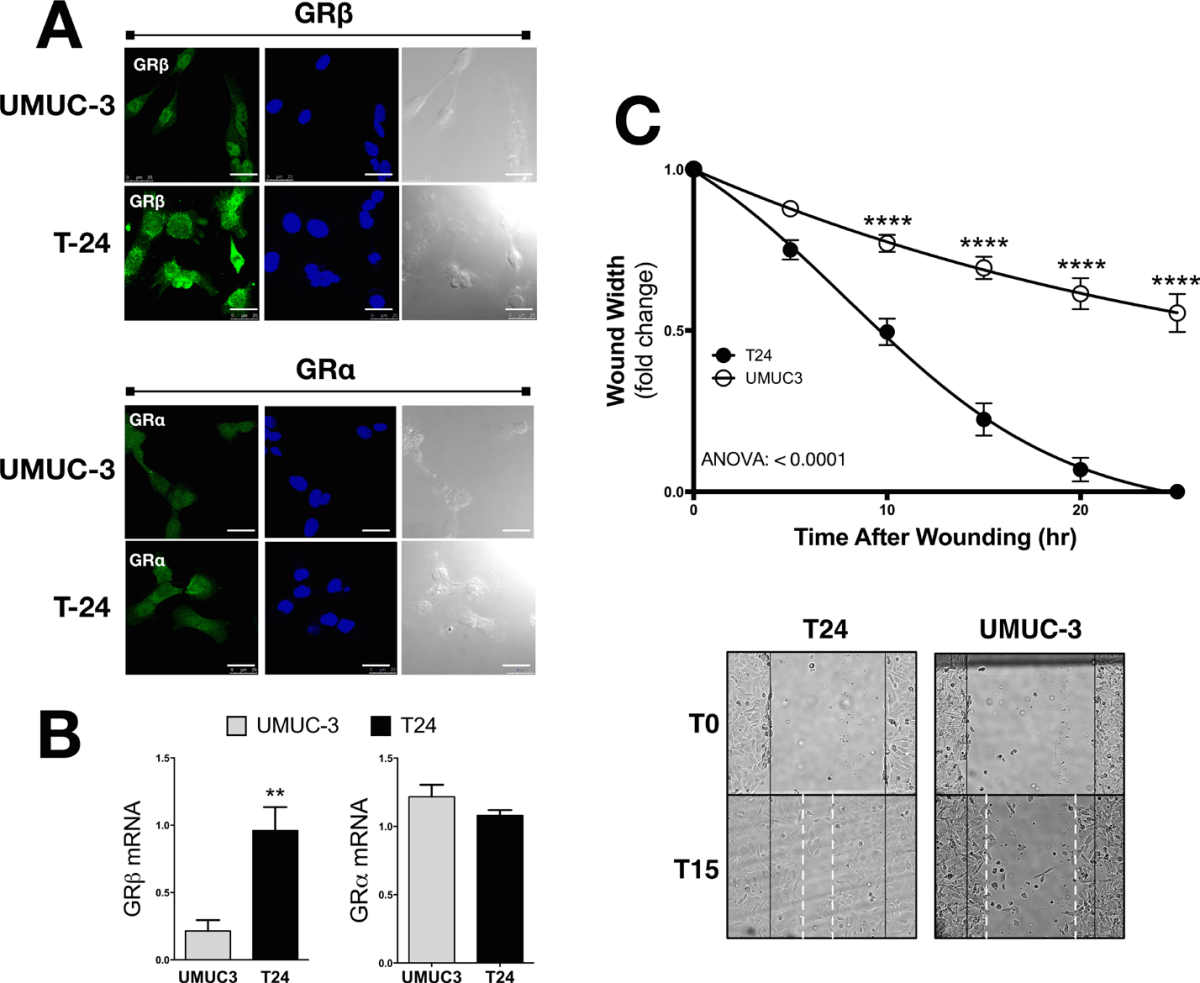
GRβ and GRα expression and migration in UMUC3 and T24 human bladder cancer cells GRβ and GRα expression was measured by immunoflourescence (**A**). Secondary antibodies (labeling GRα or GRβ) are shown in green, DAPI (nuclei labeling) are shown in blue, and a bright field images are shown in gray (scale bar = 25 μm). GRβ and GRα mRNA expression measured by Real-Time PCR (**B**). *p < 0.05; **p < 0.01 (versus UMUC3) (±S.E.; *n* = 3). Migration was measured in UMUC3 and T24 cells at 5, 10, 15, 20, and 25 hours post wounding as the fold-changed wound width remaining (**C**). Images of the migration assay at 0 and 15 hours post wounding, with wound edges marked at T0 (black solid line) and T15 (white dashed line). ANOVA *p* < 0.0001; Bonferoni comparisons ****p < 0.0001 (versus UMUC3) (±S.E.; *n* = 6).

**Figure 2:**
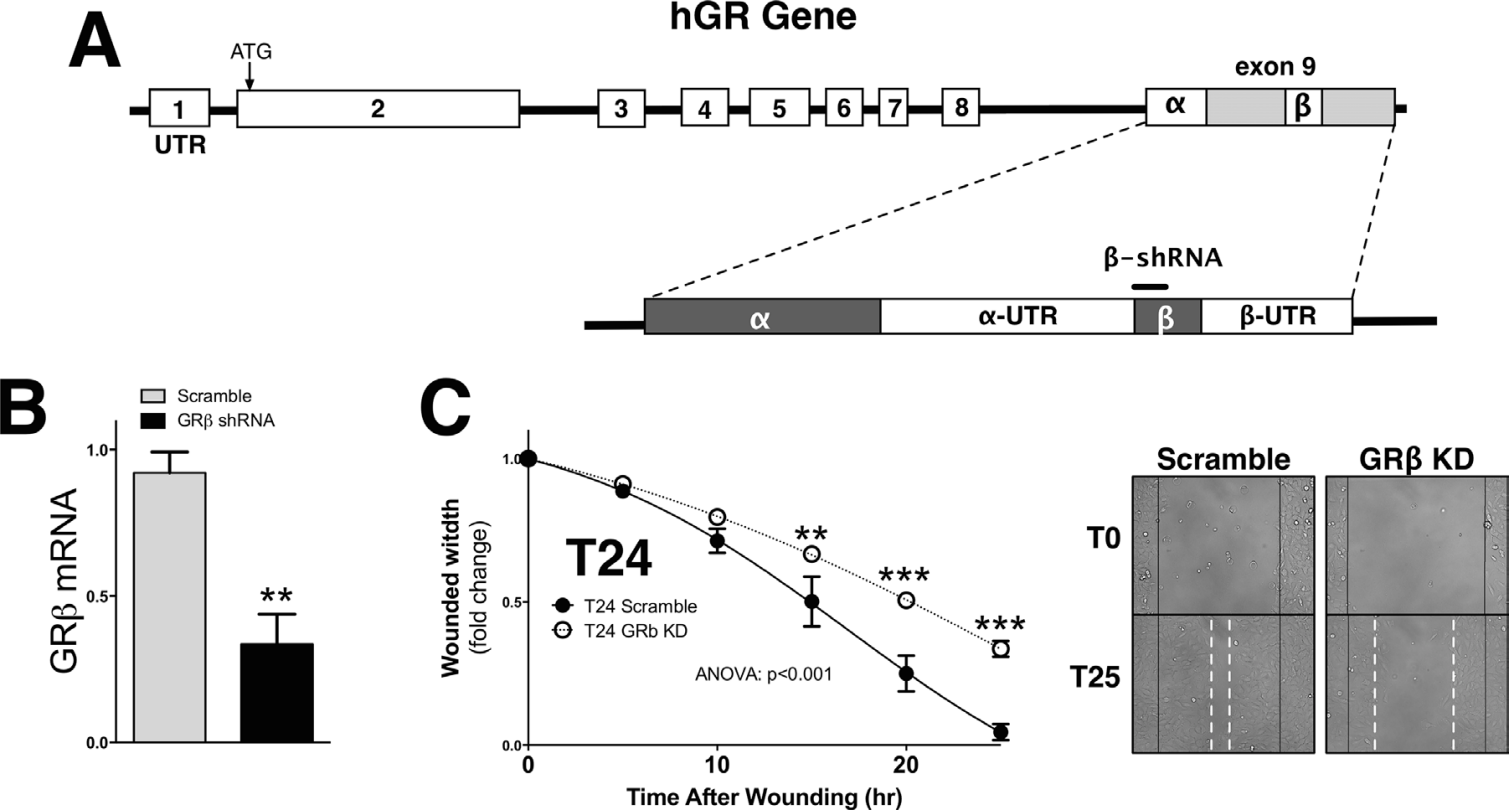
Knockdown of GRβ reduces migration of human bladder cancer cells Schematic of the the human GR gene to show the shRNA target site for GRβ (**A**). Knockdown of GRβ was confirmed by Real-Time PCR (**B**). **p < 0.01 (versus T24 Scramble) (±S.E.; *n* = 3). Migration was measured in the T24 Scramble and T24 GRβ shRNA cells at 5, 10, 15, 20, and 25 hours post wounding (**C**). Images of the migration assay at 0 and 25 hours post wounding are shown, with wound edges marked at T0 (black solid line) and T15 (white dashed line). ANOVA *p* < 0.001; Bonferoni comparisons **p < 0.01; ***p < 0.001. (*versus* T24 Scramble) (±S.E.; *n* = 6).

We have previously shown that GRβ is involved in regulating cellular pathways that are known to be involved in cancer [8]. However, the analysis was performed in non-cancerous mouse fibroblast and 3T3-L1 cells. To examine the two GR isoforms in human bladder cancer, we assayed their expression in two transitional uroepothelial cancer cell lines, UMUC3 and T24. As shown by immunofluorescence staining and mRNA expression, we found that the T24 cell line had a higher expression of GRβ compared to the UMUC-3 (Figure 1A and 1B). GRα had similar levels by immunofluorescence and mRNA in the T24 cells. To determine if human bladder cancer cells that have higher GRβ expression are more migratory, we conducted a wound-healing migration assay. The T24 cells with higher GRβ expression had a significantly (ANOVA *p* < 0.0001) faster migration compared to the UMUC-3 (Figure 1C and 1D). To show the effect of GRβ in the T24 cell line we established a stable cell line with an shRNA lentivirus targeting human GRβ (Figure 2A). The knockdown of GRβ expression in the T24 cells (64% reduction) (Figure 2B) resulted in a significant (ANOVA: *p* < 0.001) decrease in migration (Figure 2C).

### The effect of insulin and dexamethasone on GRβ & GRα in human bladder cancer cells

**Figure 3:**
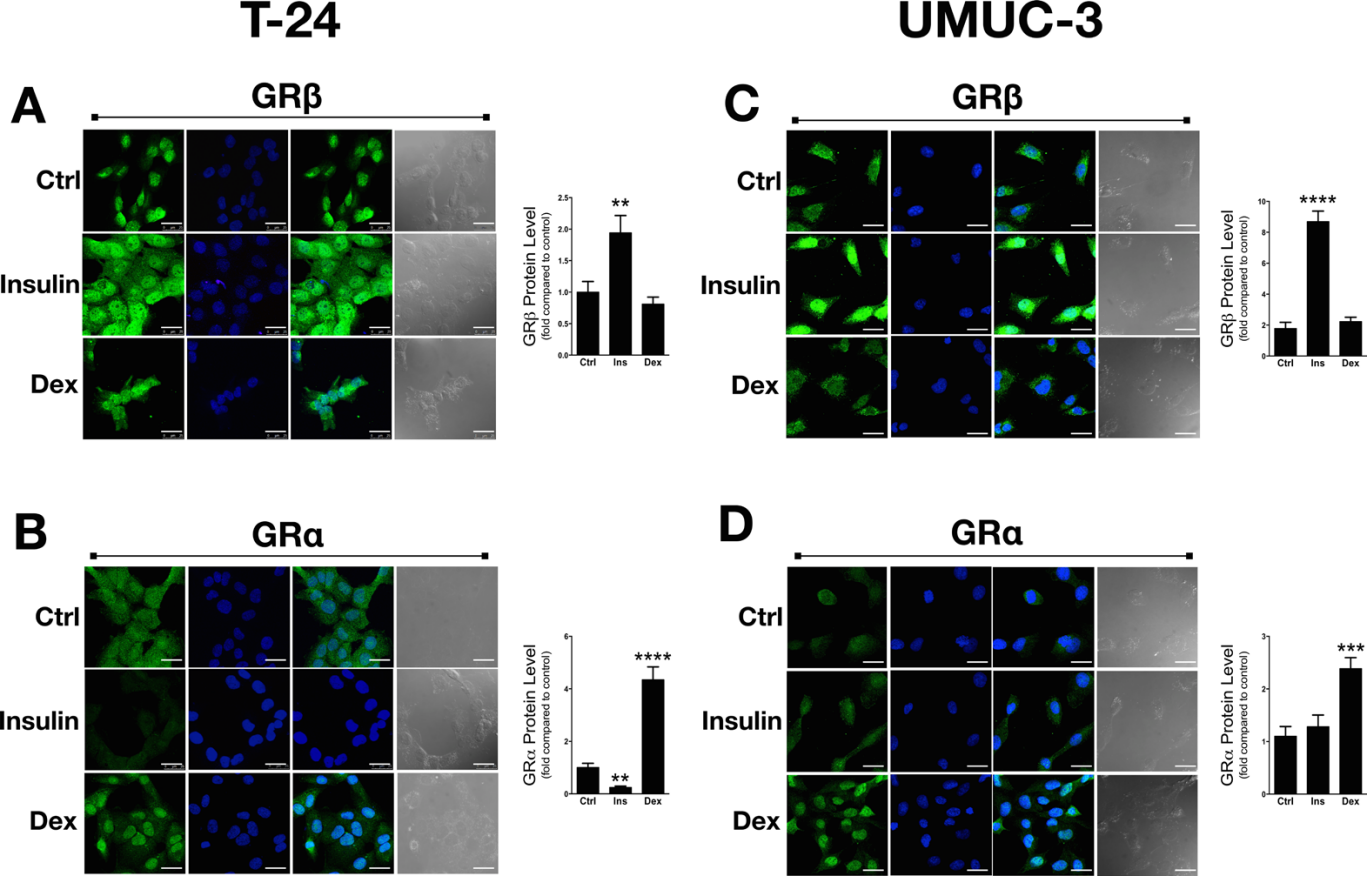
Dexamethasone and insulin treatment in T24 and UMUC-3 cells GRβ and GRα expression and location was measured using immunoflourescence with control (vehicle treatment), dexamethasone, or insulin treatments (**A–D**). Cells were seeded onto coverslips in media containing 10% dialyzed FBS for 24 hours before treating. Cells were treated with 100 nM insulin, 100 nM dexamethasone, or vehicle for 30 minutes. Secondary antibodies (labeling of human GRα or GRβ) are shown in green, DAPI (nuclei labeling) are shown in blue, a merge of the green and blue images are shown in panel 3 to represent localization, and a bright field image is shown in gray (scale bar = 25 μm). Data are represented as fold change compared to control. **p < 0.01; ***p < 0.001; ****p < 0.0001 (versus control) (±S.E.; *n* = 3).

We have previously shown that insulin increased GRβ mRNA and protein expression in cells [7, 8] and livers of mice [7]. To determine the effect of insulin or dexamethasone (Dex) on GR isoform localization and expression, we treated the T24 and UMUC-3 bladder cancer cells for 30 minutes and labeled with human GRα or GRβ antibodies for immunofluorescence staining. Insulin treatment significantly increased GRβ expression in the T24 (*p* < 0.01) and UMUC-3 (*p* < 0.0001) cells (Figure 3A and 3C). However, there was no difference observed in GRβ expression or localization in the human bladder cancer cells with Dex treatment, even though we have previously shown that GCs increased GRβ in normal mouse cells [7]. As for localization, GRβ was higher in the nucleus in the UMUC-3 with insulin, but not with Dex. There was no change in GRβ localization with insulin or Dex in the T24 cells. The GRα expression was significantly decreased (*p* < 0.001) by insulin in the T24 cells, but no effect was observed in the UMUC-3 (Figure 3B and 3D). Dex treatment increased GRα protein in both cell lines, as well as translocation from the cytoplasm (control) to the nucleus (Dex). These results may indicate a pro-growth pathway that involves the induction of GRβ and inhibition of GRα for proliferation or migration.

### GRα controlled gene transcription in human bladder cancer cells

**Figure 4:**
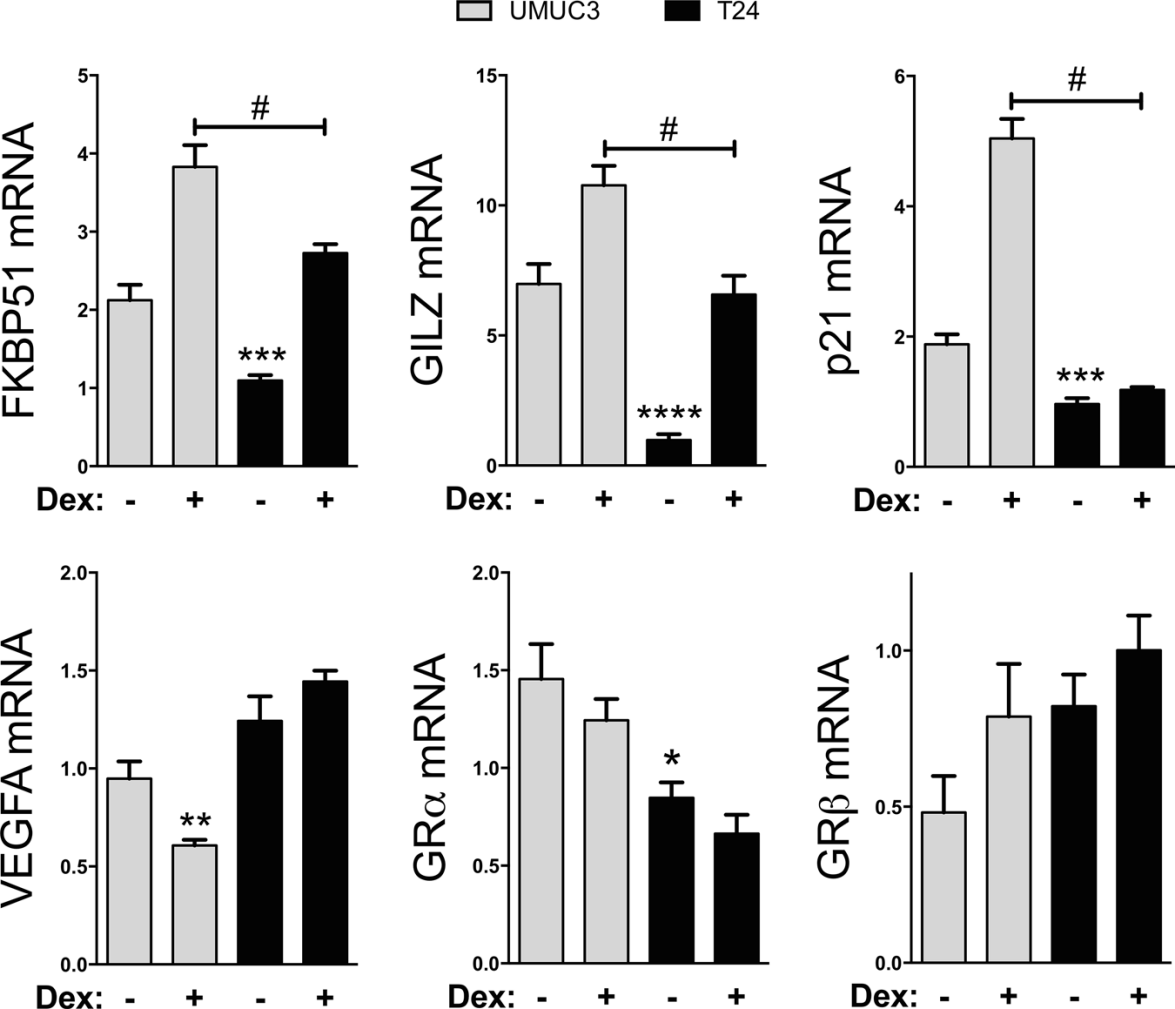
GRα controlled gene expression in T24 and UMUC-3 human bladder cancer cells GRα controlled gene response was measured by Real-time PCR for FKBP51, GILZ, p21, VEGFA, GRα, and GRβ. Cells were seeded in media containing 10% Dialyzed FBS for 24 hours before treatment. Cells were then treated with 100nM dexamethasone or vehicle for 2 hours before mRNA was harvested. *p < 0.05; **p < 0.01; ***p < 0.001; ****p < 0.0001 (versus UMUC3 control); #*p* < 0.05 (versus UMUC3 Dex) (±S.E.; *n* = 3).

The role of the GR isoforms in human bladder cancer is unknown, especially the gene regulator activity of GRα. To determine the GRα-induced gene activity in human bladder cancer cells, we treated the T24 and UMUC-3 cells with Dex for 2 hours in hormone-free dialyzed serum. To test genes that are directly regulated by GRα, we measured mRNA expression of known controlled genes FK506 binding protein 51 (FKBP51), glucocorticoid-induced leucine zipper (GILZ), and p21 (Figure 4). The UMUC3 cell line was more responsive to the Dex treatment on FKBP51, GILZ and p21 mRNA expression, likely due to the lower expression of GRβ leading to less GRα inhibition. The VEGFA expression was not different in the T24 cells, but was significantly (*p* < 0.01) deceased by Dex treatment in the UMUC-3 cells. The GRα expression was significantly (*p* < 0.05) lower in the T24 cells, but Dex treatment did not change the mRNA expression in UMUC-3 or T24 cells. The GRβ mRNA expression did not change with Dex treatments. However, it should be noted that the levels of GRβ and GRα mRNA are different than in Figure 1B, which is mostly likely due to the use of hormone-free serum for the glucocorticoid treatment.

### GRβ expression is controlled by miRNAs

**Figure 5:**
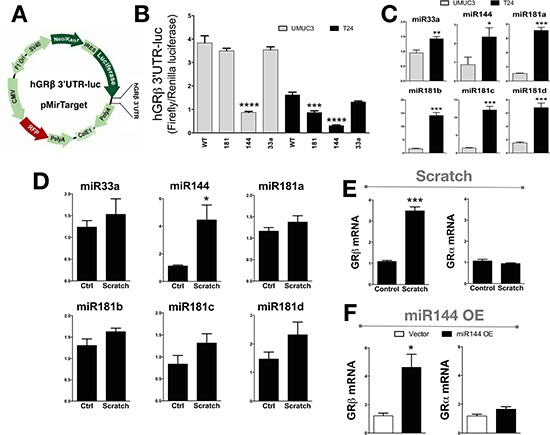
The human 3′UTR of GRβ is regulated by miR144 The pMirTarget vector containing the 3′UTR of human GRβ was cloned into a luciferase reporter gene (3′UTR GRβ-Luc) (**A**). The T24 and UMUC-3 bladder cancer cells were transfected with the 3′UTR GRβ-Luc expression construct with mutation in the miRNA binding site for miR181, miR144, or miR33a and was measured by a luciferase assay, and normalized to renilla (**B**). ***p < 0.001; ****p < 0.0001 (*versus* WT) (±S.E.; *n* = 6). The miRNA expression in the UMUC3 and T24 cells was measured using Real-Time PCR (**C**). *p < 0.05; **p < 0.01 (versus UMUC3) (±S.E.; *n* = 3). Total RNA was harvested at the time of wounding and 3 hours after from the T24 cells in media containing 10% dialyzed FBS to determine the expression during migration assay for miRNA expression (**D**). *p < 0.05 (*versus* T0) (±S.E.; *n* = 3), and for mRNA expression of GRβ and GRα expression (**E**). ***p < 0.001 (*versus* T0) (±S.E.; *n* = 3). A plasmid containing the human miR144 in the pCMV-MIR vector was transfected in the T24 cells to show how miR144 overexpression affected the expression of GRβ and GRα as measured by Real-time PCR (F). *p < 0.05 (*versus* T24 Vector) (±S.E.; *n* = 3).

To assay miRNA control of GRβ expression, we used the *in-silico* prediction software Targetscan (version 6.2) to find miRNAs that may bind to the 3′- UTR of human GRβ (Supplementary Figure 1) [37–39]. Three miRNAs were predicted to bind the 3′UTR of human GRβ (miR33a, miR181-a/b/c/d, and miR144). To determine which of the predicted miRNAs may regulate human GRβ expression, we used the pMirTarget vector with the 3′ UTR of human GRβ (pMirTarget 3′ UTR hGRβ) inserted after the luciferase reporter gene, which is under the control of the IRES promoter (Figure 5A). Next, we mutated the predicted binding sites to all adenines (Supplementary Figure 2) to determine the potential of the miRNA on human GRβ expression. We transfected the UMUC-3 and T24 cells with the pMirTarget 3′ UTR hGRβ mutants and measured luciferase activity (Figure 5B). Mutational analysis of the miR144 binding site resulted in a decrease of 77% (UMUC-3) and 81% (T24) in the reporter expression, indicating that miR144 may enhance human GRβ expression. Interestingly, mutation of the miR181 site also decreased of luciferase in pMirTarget 3′ UTR hGRβ, but this was not observed in the UMUC-3. Total RNA was extracted from the UMUC-3 and T24 cells to measure the miRNA expression that may target the 3′ UTR of human GRβ (Figure 5C). Interestingly, miR33a, miR144, miR181a, miR181b, miR181c, and miR181d were all increased in the T24 cells. Next, we wanted to determine if miR33a, miR144, miR181a, miR181b, miR181c, or miR181d changed during a scratch assay and if this affected the human GRβ or GRα expression. A scratch (wounding) assay of the T24 cell line showed that miR144 (4 fold) and GRβ (3.2 fold) were both increased (Figure 5D and 5E). The miR33a, miR181a, miR181b, miR181c, and miR181d were unchanged during the scratch (wounding) assay. Interestingly, GRα mRNA expression was also unchanged with the scratch (wounding) assay. To show that miR144 specifically regulates human GRβ, we overexpressed a plasmid containing the precursor of human miR144 in pCMV-MIR or empty vector. The miR144 containing pCMV-MIR vector resulted in a 184-fold increase in miR144 expression compared to the empty vector (*p* = 0.002). The overexpression of miR144 resulted in a significant (p < 0.05) increase in human GRβ expression (3.8 fold), while not changing GRα (Figure 5F).

### Dexamethasone control of migration and miRNA expression

**Figure 6:**
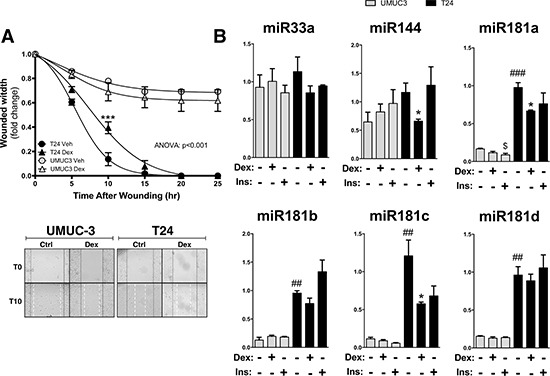
The effect of dexamethasone treatment on cell migration and miRNA expression in UMUC-3 and T24 bladder cancer cells Migration was measured in the UMUC-3 and T24 cells that were treated with either vehicle or 100 nM dexamethasone for 30 minutes prior to wounding, and is presented as the fold-change in the wound width remaining (**A**). Images of the migration assay at 0 and 10 hours post wounding are shown, with wound edges marked at T0 (black solid line) and T15 (white dashed line). ANOVA *p* < 0.001; Bonferoni comparisons ***p < 0.0001 (*versus* Vehicle) (±S.E.; *n* = 6). Total RNA was extracted from the UMUC3 and T24 cells after treating with either vehicle, 100 nM dexamethasone, or 100 nM Insulin in dialyzed media for 2 hours before harvesting, and miRNA expression was measured by Real-time PCR (**B**). *p < 0.05; (Dex versus Veh); $*p* < 0.05; (Ins versus Veh); ##*p* < 0.01; ##*p* < 0.001; (T24 *versus* UMUC3) (±S.E.; *n* = 6).

Next, we investigated the effect of Dex on migration and regulation of miRNA expression. Dex treatment was performed 30 minutes before scatch assay (wounding) of the T24 bladder cancer cells. The Dex treatment significantly (ANOVA: *p* < 0.001) decreased migration, while there was no affect in the UMUC-3 cells (Figure 6A). Dex treatment decreased expression of miR144, miR181a, and miR181c in the T24 cells, but not in the UMUC-3 cells (Figure 6B). Insulin did not significantly change expression of miR33a, miR144, miR181a, miR181b, miR181c, or miR181d in the T24 cells. However, insulin did suppress miR181a expression in the UMUC-3 cells.

### Drug targeting the miR144 enhancement of GRβ

**Figure 7:**
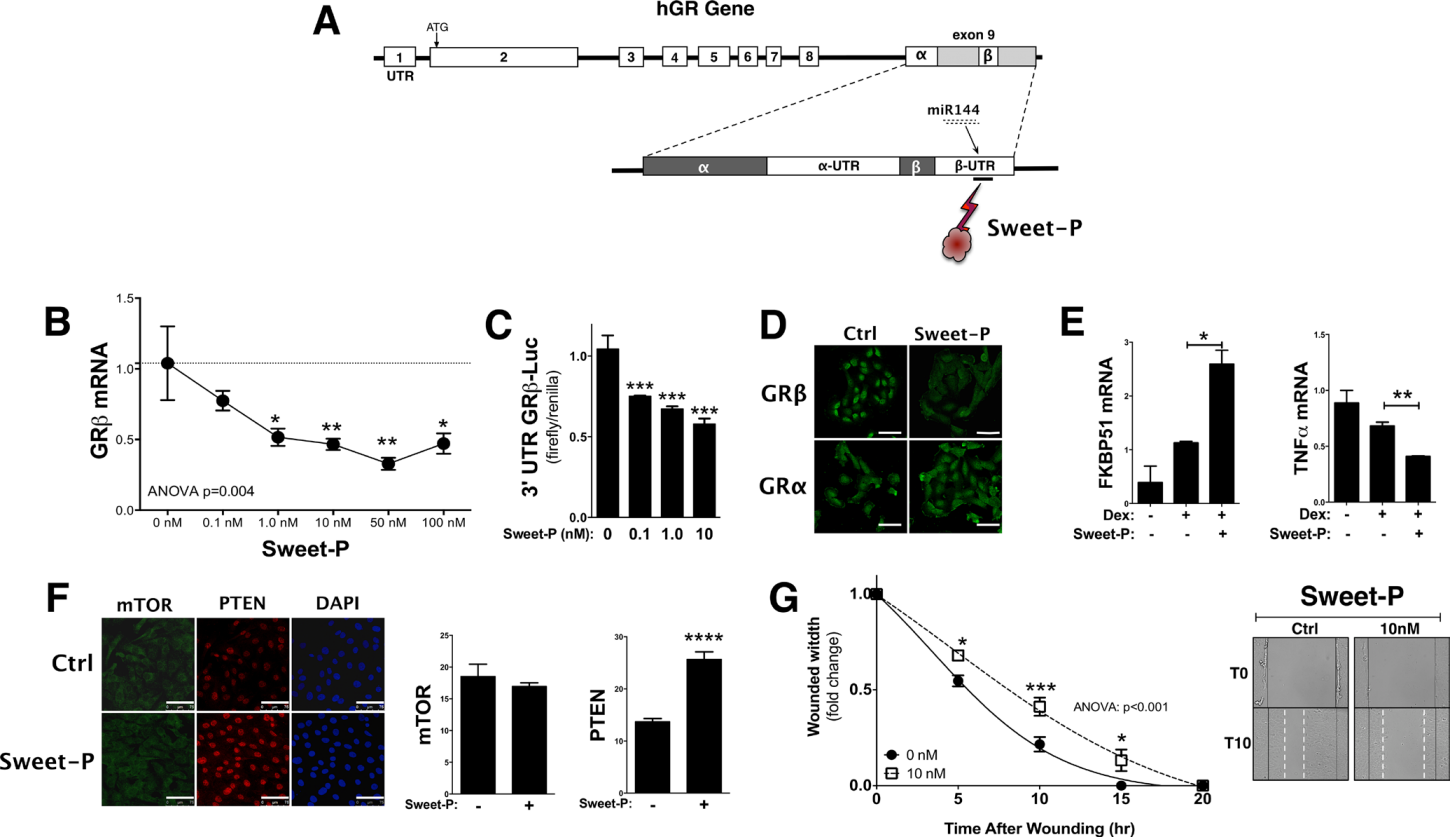
Blocking the miR144 binding site in the 3′UTR of human GRβ by Sweet-P inhibits expression and cell migration A peptide nucleic acid (PNA) conjugated to a cell penetrating peptide (CPP) (Sweet-P) was designed to bind to the miR144 binding site in the 3′UTR of human GRβ mRNA (**A**). GRβ expression in T24 cells was measured at increasing doses of Sweet-P (0, 0.1, 1.0, 10, 50, and 100 nM) for 48 hours after and human GRβ mRNA was measured by Real-time PCR (**B**). ANOVA *p* < 0.01; Dunnett's comparisons *p < 0.05; **p < 0.01 (versus 0nM) (±S.E.; *n* = 6). The 3′UTR GRβ-Luc was used to determine the dose dependence of Sweet-P (0, 0.1, 1.0, and 10 nM) by luciferase (**C**). ***p < 0.001 (*versus* 0nM) (±S.E.; *n* = 4). Immunostaing of human GRβ and GRα with 10nM Sweet-P treatment for 48 hours in T24 bladder cancer cells (**D**). Sweet-P treatment for 48 hours and then 2 hours of dexamethasone in T24 cells and Real-time PCR analysis of FKBP51 and tumor necrosis factor α (TNFα) (**E**). *p < 0.05; **p < 0.01 (versus no dex or Sweet-P control) (±S.E.; *n* = 3). Immunostaing of mTOR and PTEN with 10nM Sweet-P treatment for 48 hours in T24 bladder cancer cells (D) (scale bar = 75 μm). Migration was measured in the T24 after being treated with 10 nM Sweet-P for 48 hours prior to wounding, and is presented as the fold-change for the wound width remaining (**G**). Images of the migration assay at 0 and 10 hours post wounding are shown, with wound edges marked at T0 (black solid line) and T15 (white dashed line). ANOVA *p* < 0.001; Dunnet comparisons *p < 0.05; **p < 0.01; ***p < 0.0001 (*versus* 0nM) (±S.E.; *n* = 6).

To inhibit the binding of miR144 to the 3′UTR of GRβ, we developed a peptide nucleic acid (PNA) conjugated with a cell-penetrating peptide (CPP) (Sweet-P) targeting the site (Figure 7A). A dose dependence response curve indicated that Sweet-P significantly (*p* < 0.05) decreased GRβ mRNA expression in the T24 human bladder cancer at 1.0 nM, 10 nM, 50 nM, and 100 nM (Figure 7B). To confirm our endogenous gene finding, we transfected the T24 bladder cancer cells with the pMirTarget 3′ UTR hGRβ construct and treated with Sweet-P for 48 hours (Figure 7C). The luciferase expression of the pMirTarget 3′ UTR hGRβ construct was significantly (*p* < 0.001) reduced at 0.1 nM, 1.0 nM, and 10 nM. Sweet-P (10 nM) reduced GRβ protein expression, but had no effect on GRα (Figure 7D). Furthermore, Sweet-P significantly increased GRα activity with dexamethasone treatment by enhancing expression of FKBP51 (*p* < 0.05) and decreasing a known GRα regulated gene, tumor necrosis factor α (TNFα) (Figure 7E). To show that Sweet-P specifically targets miR144-binding site in the 3′UTR of hGRβ, we measured protein expression by immunohistochemistry of two known miR144 targets that have been shown to be suppressed, PTEN [35] and the mammalian target of rapamycin (mTOR) [40]. The results show that mTOR expression is unaffected by Sweet-P (10 nM), and PTEN expression was significantly (*p* < 0.0001) increased with treatment. To determine if Sweet-P could inhibit migration, we treated the T24 bladder cancer cells with 10 nM Sweet-P during a scratch (wounding) assay. The results show that Sweet-P significantly (ANOVA: *p* < 0.001) inhibited migration of the T24 bladder cancer cells during migration (Figure 7D).

## DISCUSSION

This is the first study to show that GRβ is enhanced during the migration of human bladder cancer cells. Suppression of GRβ by lentiviral shRNA decreased the migration of T24 cells. Yin et al. showed that GRβ increases the migration of astrocytes and brain cancer cells (glioblastoma) [22]. Several other studies have also demonstrated that GRβ is elevated in cancers and inflammatory diseases, which leads to increased growth [20, 41–46]. GCs have been shown to inhibit migration and proliferation of cancer cells in medulloblastoma [47], osteosarcomas [48, 49], A549 human lung cancer cells [50], as well as other lung cancer cells: squamous cell carcinoma lines (EPLC-32M1 and NCI-H157), large-cell carcinoma cell line (LCLC-97TM1) and a cell line from mesothelioma (MSTO-211H) [51]. Increasing GRβ may provide a state of GC resistance that reduces their ability to inhibit growth and migration. Bombesin has been shown to induce resistance to GCs by induction of GRβ in human prostate cancer cells [43]. We showed in this study that the T24 human bladder cancer cells had a reduced response to GCs compared to the UMUC-3, which may be due to elevated GRβ. The migratory potential of T24 cells, but not the UMUC3 cells, was inhibited by dexamethasone. Dexamethasone has been previously shown to inhibit the invasion of bladder cancer cells, including the UMUC-3 [18]. In this study, we show that dexamethasone treatment inhibited miR144, which we have shown is a positive regulator of GRβ and is increased during migration. The inhibition of miR144 may be a potential mechanism that migration was reduced by dexamethasone in the T24 cells.

As shown by the mutation in the 3′UTR of human GRβ reporter, and plasmid overexpression, miR144 is a positive regulator of human GRβ expression and not GRα. Iwaya et al. showed that miR144 downregulated mTOR, a regulator of cellular growth and metabolism, and the loss of miR144 leads to the progression of colorectal cancer [40]. Zhang et al. showed that miR144 downregulated PTEN expression [35], a tumor suppressor gene that regulates many cellular functions including cell proliferation. Guo et al. showed that miR144 inhibits bladder cancer proliferation by targeting the enhancer the zeste homolog 2 (EZH2), a downstream regulator of the Wnt/β-catenin pathway that mediates growth [34]. However, the effects of miR144 on migration were not tested. There are a plethora of targets for miRNAs, and the specific blockade of miR144 binding to the 3′UTR of GRβ by Sweet-P resulted in decreased GRβ mRNA expression and 3′UTR GRβ-luc reporter assay. The effect of Sweet-P was specific for the 3′UTR of human GRβ, as mTOR and PTEN, which are known to be suppressed by miR144, were not lower but PTEN was significantly higher. We have recently shown that GRβ binds to the PTEN promoter to inhibit expression [8], and therefore the suppression of GRβ by Sweet-P treatment caused derepression of PTEN. Sweet-P did suppress GRβ protein expression, but did not change GRα. However, the GRα activity was increased with Sweet-P treatment. Moreover, the downregulation of GRβ by Sweet-P inhibited migration of bladder cancer cells, indicating that it may serve as a potential therapy for bladder cancer. The inhibitory effect of dexamethasone on the migration of the T24 cells supports that GRα is a suppressor of bladder cancer, which is also shown by Sweet-P enhancing GRα activity and suppressing migration of the bladder cancer cells. Dexamethasone inhibition of VEGF-A supports that GCs may inhibit bladder cancer, VEGF-A levels were found to be greater in higher-grade urothelial tumors [52]. Dexamethasone decreased miR144 and migration of the T24 cells, which indicates that suppression of miR144 levels may also reduce GRβ expression. However, two-hour dexamethasone treatment in hormone-free serum did not affect GRβ mRNA, or 30-minute treatment did not change the protein. Glucocorticoids may alter GRβ expression with longer treatment. We showed in mouse fibroblast that GRβ increased with dexamethasone treatment [7], but no change was observed in mouse C2C12 myoblast [53]. During migration, dexamethasone suppression of miR144 may have a larger impact on GRβ expression.

The effect of insulin on enhancing GRβ expression and inhibiting GRα suggests that it may increase the risk of bladder cancer. However, insulin did not increase miR144 expression with an acute two-hour treatment, which suggests that insulin may enhance GRβ levels by a different mechanism. The effect of insulin on cancer cell migration rate has not been studied, but the insulin-like growth factor receptor I (IGFR-I) has been shown to promote invasion of bladder cancer cells through an Akt and mitogen activated protein kinase (MAPK) dependent mechanism [54]. There has been no correlation found for bladder cancer in insulin-resistant type II diabetics [55], which suggests that insulin may not have a role. However, IGF-I may signal to GRβ to increase bladder cancer invasion, but the effect of IGF-I on GRβ expression has not been investigated. Most likely the increase of miR144 during T24 migration and its enhancement of GRβ expression are mediated in a non-insulin dependent manner. Two drugs for the treatment of type II diabetes, rosiglitazone and pioglitazone, have been shown to induce bladder cancer [56, 57], but their effect on miR144 or GRβ expression is unknown. Recent studies have revealed that there is a 3:1 incidence in men compared to women for bladder cancer, which may be mediated by the androgen receptor (AR) [4]. Presumably, there may be an interaction between AR and GRβ in prostate cancer. Ligr et al. showed that suppression of GRβ in LNCaP, RC165, and DU145 human prostate cancer cells inhibited growth [58]. However, the signaling involvement of GRβ and AR in bladder cancer has not been investigated.

In conclusion, GRβ mediates bladder cancer migration and may serve as a target for therapy. The 3′ UTR of GRβ is enhanced by miR144 during human bladder cancer migration. Blocking the interaction of miR144 with the 3′ UTR of GRβ by Sweet-P slowed bladder cancer migration. The antagonism of human GRβ by Sweet-P, drug interaction, or gene targeting may serve as a potential treatment for bladder cancer.

## MATERIALS AND METHODS

### Cell lines and culture

The human uroepithelial carcinoma cell lines UMUC-3 and T24 (ATCC) were routinely cultured and maintained in Minimum Essential Medium (MEM) containing 10% fetal bovine serum (FBS) with 1% antibiotic-antimycotic. Cells were maintained at 37°C and 5% CO^2^. Media was changed to MEM containing 10% dialyzed-FBS with 1% antibiotic-antimycotic 24 hours before hormone treatments.

### RNA extraction for mRNA quantification and real-time PCR analysis

Total RNA was extracted from cell cultures using the 5-Prime PerfectPure RNA Cell Kit (Fisher Scientific Company, LLC). Total RNA was read on a Nano Drop 2000 spectrophotometer (Thermo Fisher Scientific, Wilmington, DE) and cDNA was synthesized using High Capacity cDNA Reverse Transcription Kit (Applied Biosystems). PCR amplification of the cDNA was performed by quantitative real-time PCR using TrueAmp SYBR Green qPCR SuperMix (Smart Bioscience). The thermocycling protocol consisted of 10 min at 95°C, 40 cycles of 15 sec at 95°C, 30 sec at 60°C, and 20 sec at 72°C and finished with a melting curve ranging from 60–95°C to allow distinction of specific products. Normalization was performed in separate reactions with primers to GAPDH.

### RNA extraction for miRNA quantification and real-time PCR analysis

Total RNA was extracted from cell cultures using the miRNeasy Mini Kit (Qiagen). Total RNA was read on a Nano Drop 2000 spectrophotometer (Thermo Fisher Scientific, Wilmington, DE) and cDNA was synthesized using the miScript II RT Kit (Qiagen). PCR amplification of the cDNA was performed by quantitative real-time PCR using miScript SYBR Green PCR Kit (Qiagen). The thermocycling protocol consisted of 15 min at 95°C, 40 cycles of 15 sec at 94°C, 30 sec at 55°C, and 30 sec at 70°C and finished with a melting curve ranging from 60–95°C to allow distinction of specific products. The miScript Primer Assay primers were purchased from Qiagen. Normalization was performed in separate reactions with primers to Hs_RNU6-2_11, At_U19_1, and Hs_SNORD61_11.

### Immunoflourescence and microscopy

Samples were imaged using a Leica TCS SP5 laser scanning confocal microscope (Leica Microsystems, Bannockburn, IL) equipped with conventional solid state and a Ti-sapphire tunable multiphoton laser (Coherent, Santa Clara, CA). Images were acquired in the XYZ plane in 1 μm steps with a 63X oil objective (NA 1.40). Images were acquired with the LAS AF software in sequential scan mode. Alexa Fluor 488 was excited at 488 nm with collection at 500–558 nm and DAPI was excited with the multi-photon (MP) laser tuned to 790 nm with collection at 420–500 nm. Images are 2D projections of the of image stack as labeled. Three images were taken per slide and ImageJ software was used to measure the immunofluorescence of each cell (average 40 cells) in the images.

### Migration assay

Cells were seeded on a 6-well plate and grown for 24 hours until a monolayer of 90% confluent cells were obtained. A scratch wound in the cell monolayer was introduced using a sterile pipette tip. Images were taken at the time of wounding, and every 5 hours thereafter. Migration was measured as the fold change of the width of the wound remaining. To determine miRNA and mRNA expression during the scratch (wounding assay), we collected total RNA using the miRNeasy Mini Kit (Qiagen) (described above) at 0 and 3 hours after the migration.

### Generation of lentiviral constructs

To establish a T24 cell line that has hGRβ stably knocked down, the pGFP-C-shLenti plasmid containing either GRβ shRNA (CCAGAAAGCACATCTCACACATTAATCTG) or scrambled shRNA (Origene) was packaged into a lentiviral construct using the Lenti-vpak Packaging Kit (Origene) by transfection in 293-GP2 cells. The supernatants were harvested and the cell debris was removed by filtration through 0.45 μM filter. The supernatant was used to infect T24 cells after addition of polybrene (10 ug/ml, Sigma Chemical Co., St. Louis, MO) to establish cell lines with stable expression of hGRβ shRNA (T24 GRβ KD) or expressing scrambled shRNA (T24 Scramble). After 72 h the cells were initially selected using Puromycin (10 μg/mL). Cells were then secondarily selected by sorting through flow cytometry for GFP by the Flow Cytometry Core Facility at the University of Toledo Health Science Campus.

### Transient transfection

For transient transfection cells were plated on a 6-well or 12-well dish in MEM containing 10% FBS. Cells were washed with OPTI-MEM and transfected using GeneFect (Alkali Scientific, Inc.), according to the manufacturer's protocol. OPTI-MEM was removed after 12 h and MEM containing 10% FBS was added.

### Promoter reporter assays

The expression vector pMirTarget containing the 3′UTR of hGRβ (hGRβ 3′UTR-luc) was purchased from (Origene). The binding sites were mutated using the Quik Change Lightning Multi Site-Directed Mutagenesis Kits (Agilent Technologies). Successful mutations were confirmed through sequencing by Operon MWG. Cells were seeded onto a 12-well plate and grown overnight. Transient transfection was performed as described above. To determine the effect of Sweet-P on the T24 bladder cancer cells, we treated 24 h after transfection for 48 hours post transfection, 3′UTR GRβ-Luc WT or mutant expression was measured by luciferase, and pRL-CMV Renilla reporter for normalization to transfection efficiency, using the Promega dual luciferase assay system (Promega, Madison, WI).

### miRNA overexpression

The cloning vector pCMV-MIR containing the miR144 sequence was purchased from Origene. Cells were seeded on a 6-well plate, and transient transfection of the plasmid was completed as above. After 48 hours-post transfection, RNA was harvested as described above.

### Targeting of the human GRβ mRNA

A peptide nucleic acid (PNA) conjugated to a cell penetrating peptide (CPP) targeting the miR144 binding site in the 3′UTR of the human GRβ (Sweet-P) was designed using PANAGENE website (http://www.panagene.com). The Sweet-P sequence targeted the miR144 binding site in the 3′ UTR of human GRβ (Supplementary Figure 2). All PNAs were attached to an O Linker and a modified TAT protein (VQRKRQKLMP) for delivery into the cell (CPP). Treatment with Sweet-P was performed for 48 hrs before the cells were analyzed.

### Statistical analysis

Data were analyzed with Prism 5 (GraphPad Software, San Diego, CA) using analysis of variance combined with Tukey's post-test to compare pairs of group means or unpaired *t* tests. Additionally, two-way ANOVA was utilized in multiple comparisons, and followed by either the Bonferroni or Dunnet post hoc analyses to identify interactions. *p* values of 0.05 or smaller were considered statistically significant.

## SUPPLEMENTARY MATERIALS FIGURES


