# Supplementary material for: Glucocorticoid receptor beta increases migration of human bladder cancer cells
Source: Oncotarget. 2016 Mar 27;7(19):27313–24. doi: 10.18632/oncotarget.8430 (PMC5053652; doi:10.18632/oncotarget.8430)
Supplement: Supplementary file 1 [file oncotarget-07-27313-s001.pdf]

Glucocorticoid receptor beta increases migration of human bladder cancer cells

Supplementary Materials

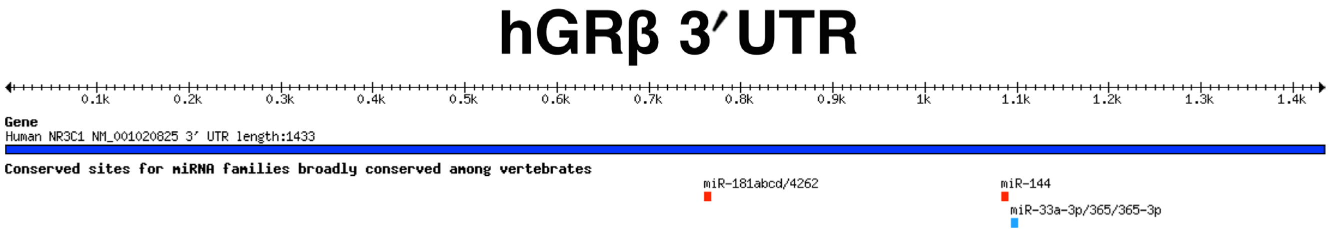

Supplementary Figure S1: *In-silico* analysis of the 3′UTR of human GRβ. The human GRβ 3′UTR analysis of potential miRNA binding sites using human TargetScan (version 6.2) software.

3′UTR of human GRβ (with TGA stop codon)

TGAttttcatcccaacaatcttggcgctcaaaaaatagaactcaatgagaaaaagaagattatgtgcactt
cgttgtcaataataagtcaactgatgctcatcgacaactataggaggcttttcattaaatgggaaaagaag
ctgtgcccttttaggatacgtgggggaaaagaaagtcatcttaattatgtttaattgtggatttaagtgct
atatggtggtgctgtttgaaagcagatttatttcctatgtatgtgttatctggccatcccaacccaaactg
ttgaagtttgtagtaacttcagtgagagttggttactcacaacaaatcctgaaaagtatttttagtgtttg
taggtattctgtgggatactatacaagcagaactgaggcacttaggacataacacttttggggtatatata
tccaaatgcctaaaactatgggaggaaaccttggccaccccaaaaggaaaactaacatgatttgtgtctat
gaagtgctggataattagcatgggatgagctctgggcatgccatgaaggaaagccacgctcccttcagaat
tcagaggcagggagcaattccagtttcacctaagtctcataattttagttccctttttaaaaaccctgaaaa
ctacatcaccatggaatgaaaaatattgttatacaatacattgatctgtcaaacttccagaaccatggtag
ccttcagtgagatttccatcttggctggtcactccctgactgtagctgtaggtgaatgtgttttttgtgtgt
gtgtgtctggttttagtgtcagaagggaaataaaagtgtaaggaggacactttaaaccctttgggtggagt
ttcgtaatttcccagactattttcaagcaacctggtccacccaggattagtgaccaggttttcaggaaagg
atttgcttctctctagaaaatgtctgaaaggattttattttctgatgaaaggctgtatgaaaataccctcc
tcaaataacttgcttaactacatatagattcaagtgtgtcaatattctattttgtatattaaatgctatat
aatggggacaaatctatattatactgtgtatggcattattaagaagctttttcattatttttttatcacagt
aattttaaaatgtgtaaaaattaaaaccagtgactcctgtttaaaaataaaagttgtagtttttttattcat
gctgaataataatctgtagttaaaaaaaaagtgtctttttttacctacgcagtgaaatgtcagactgtaaaac
cttgtgtggaaatgtttaacttttatttttttcatttaaatttgctgttctggtattaccaaaccacacatt
tgtaccgaattggcagtaaatgttagccatttacagcaatgccaaatatggagaaacatcataataaaaaa
atctgctttttcatta

miR33a
target sequence: GGCATTA
mutant sequence: AAAAAAA

miR144
target sequence: ATACTGT
mutant sequence: AAAAAAA

miR181
sequence: gtgaatgtg
mutate to: AAAAAAAAA

Supplementary Figure S2: The 3′UTR of human GRβ with the mutation of the binding sites for miRNAs. The sequence is shown, with the miR181a, b, c, & d binding site highlighted in green, miR144 binding site highlighted in blue, and the miR33a binding site highlighted in red.
